# Collagen binding properties separate two functionally distinct subpopulations of milk extracellular vesicles regarding bone regenerative capacity

**DOI:** 10.1016/j.mtbio.2025.102115

**Published:** 2025-07-18

**Authors:** Peng Wang, Yang Zhang, Onno J. Arntz, Marina C. Oliveira, Taozhao Yu, Zhihua Yang, Peter M. van der Kraan, Jeroen J.J.P. van den Beucken, Fons A.J. van de Loo

**Affiliations:** a Department of Experimental Rheumatology, Radboud University Medical Center, Nijmegen, the Netherlands; b Department of Dentistry–Regenerative Biomaterials, Radboud University Medical Center, Nijmegen, the Netherlands; c Radboud Institute for Medical Innovations, Nijmegen, the Netherlands; d School of Dentistry, Health Science Center, Shenzhen University, Shenzhen, China; e Immunometabolism, Department of Nutrition, Nursing School, Universidade Federal de Minas Gerais, Belo Horizonte, Minas Gerais, Brazil

**Keywords:** Extracellular vesicles, Interactome, Extracellular matrix, Bone defects

## Abstract

Extracellular vesicles (EVs) are heterogeneous in their composition. The proteins on their surface determine their binding properties to the meshwork of extracellular matrix (ECM). Here, we report that type I collagen-binding property separates two subpopulations of EVs from cow milk (mEVs): collagen-binding mEVs (^cb^^+^mEVs) and non-collagen binding mEVs (^cb-^mEVs). ^cb^^+^mEVs showed noticeable uptake by human bone marrow mesenchymal stromal cells (hBMSCs) and osteogenic functionality *in vitro* (1.2-fold increase in mineralization). By proteomics profiling we identified Annexin V (AnxV) and confirmed its enrichment on CD9 positive ^cb^^+^mEVs using immunomagnetic separation. By implanting a hydrogel construct enriched with ^cb^^+^mEVs into a femoral condyle defect in osteoporotic rats, we demonstrated their superior bone regenerative capacity *in vivo* (2.4-fold increase in bone formation). Our study suggests that EV binding to the ECM protein type I collagen can be used to isolate a functional mEV subpopulation for bone tissue regeneration. This approach represents an important step forward in relating EV properties to their functionality, which will promote clinical translation.

## Graphical abstract

**Figure:**
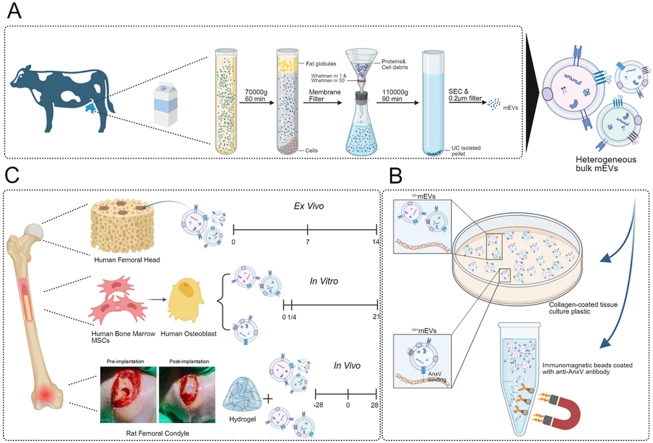

## 1 Introduction

Osteoporotic fractures are often accompanied by bone defects due to changes in bone microarchitecture, decreasing deposits of bone mineral and bone matrix components, sparseness of bone trabeculae, decreased bone strength, and increased bone fragility [1]. A bone defect is defined as a significant disruption in the integrity of bone tissue [2,3]. Globally, fracture-related bone defects such as non-unions and segmental bone defects are significant public health issues. There are approximately 178 million new bone fractures worldwide every year, with a substantial increase in prevalence since 1990 [4]. Bone defects and related conditions can lead to long-term disability, affecting a patient's ability to work and participate in daily activities and reducing the quality of life [5]. Moreover, the treatment of bone defects in the United States incurs an annual cost of approximately $5 billion, with significant expenses related to bone grafts and surgical interventions for non-unions and other complications [6]. The current standard for treating osteoporotic bone defects primarily involves surgical interventions that use autologous bone grafts to fill the defects [7], which are considered the gold standard due to their osteoinductive and osteogenic properties. However, this method is not without limitations, including donor site morbidity, limited supply, and variable clinical outcomes [8]. As for allografts and xenografts, they offer key advantages such as wide availability, ease of use, and avoidance of donor-site morbidity [9]. While they lack osteogenic potential, they provide an effective osteoconductive scaffold and, in some cases (e.g., Demineralized Bone Matrix), limited osteoinductive capacity. Their ready-to-use nature makes them valuable alternatives when autografts are not feasible [10].

Emerging cutting-edge therapies in bone tissue engineering and regenerative medicine such as using cells predominantly mesenchymal stromal cells (MSCs), growth factors and extracellular vesicles (EVs) in scaffolds are being developed to address these limitations and improve patient outcomes [11,12]. EVs are cell-secreted lipid bilayer vesicles that consist of sterols, membrane proteins and encloses an aqueous solution containing proteins, nucleic acids, and metabolites capable of influencing biological functions [13]. EVs are non-tumorigenic, biocompatible, and can be stored and transported more easily, reducing risks associated with cell-based therapies such as immune rejection and tumor formation. Additionally, compared to growth factor-based therapies that are often limited to specific signaling pathways and may require repeated administration due to their short half-lives, the versatility and stability of EVs makes EVs suitable for a wide range of therapeutic applications, including drug delivery and tissue repair [14,15].

EVs play a crucial role in cellular communication during bone remodeling [16]. Furuta et al. demonstrated that CD9-knockout mice showed impaired femur fracture healing due to reduced exosome secretion, emphasizing the significance of EVs in bone regeneration [17]. For therapeutic purposes, EVs can be isolated from cell cultures or body fluids [18]. Specifically, EVs derived from MSCs act as key posttranscriptional regulators of osteoblasts. In return, osteoblasts communicate with MSCs via EVs, creating a positive feedback loop to stimulate osteogenesis [19]. However, the main concern of enrichment of MSC-EVs from cell culture media is the contamination with exogenous EVs present in fetal bovine serum (FBS). The presence of FBS-EVs may confound the therapeutic or diagnostic analyses of EVs derived from cultured cells [20].

Recent work reported that the BMP2 and MAPK signaling pathways are crucial for the osteogenic effects of human milk derived EVs. These pathways facilitate the differentiation and mineralization of osteoblasts, thereby promoting bone regeneration [21]. While the use of human milk derived EVs faces limitations due to ethical problems, limited resources, individual heterogeneity that limit scalability and standardization of the EV product which complicates their widespread therapeutic use [22,23]. Cow milk EVs (mEVs) are known for their species cross-reactivity [24], and robust stability as they can survive the gastrointestinal tract's harsh conditions, making them effective carriers for bioactive molecules and potential drug delivery systems [25]. The scalable isolation of high-quality purified mEVs has been achieved through novel purification methods, which ensure minimal contamination from milk proteins, thus paving the way for their industrial-scale application [26]. Our previous work demonstrated that mEVs regulate bone homeostasis through influencing the activity of osteoblasts and osteoclasts [27,28]. Moreover, mEVs showed systemic osteoprotective properties in an ovariectomized (OVX) bone loss mouse model and a high-refined carbohydrate diet-induced obesity mouse model via RANKL/OPG regulation [29,30]. Recent work demonstrated that the local delivery of mEVs promoted bone repair using a mice skull defect model [31]. *Dong* et al. revealed the mechanism behind the osteogenic functionality of bulk mEVs involves the upregulation of the osteogenic gene GJA1 through the transcription factor AP3B1, providing valuable insights into how mEVs could be utilized in bone regeneration therapies.

Despite the involvement of EVs in regulating bone metabolism, functional differences among EV subpopulations, based on their inherent features such as physical characteristics and molecular cargos, present challenges for clinical translation [32]. EV heterogeneity implies that individual vesicles lack few or all of the chemical or physical characteristics of the entire population, meaning that they lack the functional properties attributed to the bulk [33,34]. This acts as a double-edged sword: it provides functional versatility, enabling EVs to perform diverse therapeutic roles and target multiple tissues, which enhances their potential in personalized medicine. Additionally, this variability complicates standardization, reproducibility, and regulatory approval, as only a subset of EVs may be bioactive while others remain inert or even counterproductive. Therefore, to validate the quality of EV preparations for therapeutic applications, the EV-inherent features could be harnessed to qualify them as suitable for subsequent clinical functional testing applications [35]. The common EV categorization is by size range, which classifies EVs as exosomes (∼30–150 nm) or microvesicles (∼100–1000 nm) [36]. Some functional aspects of EVs are known to vary with size [37,38]. For instance, recent work demonstrated that by size exclusion chromatography (SEC) three cardiac progenitor cell-EVs subpopulations with different sizes were isolated and that these showed distinct differential effects on angiogenesis [39]. In this study, we investigated small mEVs (<200 nm), separated from the larger particles by using a standard 0.2 μm filter after SEC purification [40]. Additionally, the variation in the composition of proteins on EV surface has attracted attention, as it may play a key role in determining the functional subpopulations of EVs [41]. As the size decreases, the dynamic changes in the surface-to-volume ratio make small EVs more surface-active than luminal-active [42]. Therefore, tackling EV heterogeneity and isolating the optimal EV subpopulation are supposed to be realized through utilizing surface biomolecules present on EVs [43,44]. Typically, matrix vesicles (MVs) represent one subset of small EVs released by osteoblasts, chondrocytes, or odontoblasts onto which phosphatidylserine (PS) is highly abundant on the outside the membrane. It has been reported that via the high affinity of PS for calcium ions, MVs can adhere to the extracellular matrix (ECM) by allowing calcium ions to form a bridge between the PS on the MV surface and negatively charged ECM [45].

Emerging data demonstrated that EVs actively interact with ECM components, including collagen, laminin, and fibronectin through adhesion molecules expressed on their surface [46]. The molecular foundation of the interactions between EVs and ECM components is anticipated to depend on their biochemical composition and chemical bonds. Specifically, hydrogen bonds have been identified as linking certain subsets of EVs with fibronectin or collagen in the heparin-binding domain, while other subsets of EVs possess exposed cysteines that form covalent bonds with ECM proteins like laminin [47]. Understanding the interaction of the EV surface with the biological environment is essential to fully harness the potential of EVs for applications [48]. For example, recent work observed that the migration of EVs derived from tumors cells through the interstitium depends on the presence of integrin α3β1 and α6β1 on their surface, which contribute to their binding potential to laminin-rich ECM [49]. This integrin-mediated ECM binding influences the population of EVs entering blood and lymphatic capillaries and further determines the spatial distribution of bound and free EVs within the interstitium. In bone ECM, the predominant protein is type I collagen, which plays an important role in bone regeneration [50]. During the process of new bone formation, type I collagen secreted by osteoblasts serves as a template to initiate and propagate mineralization [51]. Previous work showed that identifying an active collagen-binding domain from bioactive proteins can offer a novel approach for targeted osteogenic differentiation [52]. Specifically, *Choi* et al. investigated the interaction between binding peptide and collagen matrix activates the ERK1/2 and Akt signaling pathways and increases osteoblastic differentiation in human osteosarcoma cells. *In vivo*, a hydroxyapatite scaffold modified with the peptide significantly improved bone formation in a rabbit calvarial defect model, highlighting its potential as a bioactive agent for bone tissue engineering [53]. It has been demonstrated that the binding sites of collagen for artificial peptide present on the surface of MSC-EVs enhance EVs retention and further accelerate tissue regeneration [54]. Considering EV heterogeneity hence we regard collagen-binding capacity as an eminent “EV-inherent feature” regarding the osteogenic functionality of an EV subpopulation.

We herein demonstrate the osteogenic functionality of bulk mEVs using *in vitro* MSC-osteogenic differentiation and *ex vivo* human bone organ culture models. Through harnessing collagen binding properties, we next separate two subpopulations of mEVs: collagen-binding mEVs (^cb^^+^mEVs) and non-collagen binding mEVs (^cb-^mEVs) and demonstrate their distinct functional properties regarding osteogenic functionality *in vitro*. By proteomic profiling, we identify Annexin V (AnxV) and show its role in ^cb^^+^mEV subpopulation-induced osteogenesis. Most importantly, we reveal that ^cb^^+^mEVs enriched for AnxV represent the functional mEV subpopulation that promotes bone defect repair under osteoporotic conditions *in vivo*.

## 2 Materials and methods

### 2.1 Cow milk extracellular vesicles isolation

mEVs were isolated from commercial semi-skimmed milk derived from Holstein-Friesian cows (breed of large dairy cattle originating in northern Holland) as previously described [55]. Briefly, the milk was centrifuged for 60 min at 70.000 g at 4 °C in a SW60 rotor (Beckman Coulter). Afterwards, the fat layer and cells were removed, and supernatant was collected and filtered with Whatman nr 1 and Whatman nr 50. Thereafter the samples were centrifuged again for 90 min 110,000 g at 4 °C. After discarding the supernatant, PBS was added to dissolve the pellets, which were then filtered using a 0.22 μm filter and stored at 4 °C for future use. Thereafter, the subsequent SEC was utilized to remove the co-isolated protein contamination. In short, sepharose CL-2b (Cytiva; GE17-0140-01) was stacked in a 10 ml syringe (56 mm). mEVs (500 μL) were loaded on top of the column and eluted with PBS, and fractions 4 and 5 were collected. After SEC, the samples were filtered in 0.22 μm filter and were stored at 4 °C until further use.

### 2.2 Cow milk extracellular vesicles characterization

The concentration and size distribution of mEVs were determined using the NanoSight Tracking Analysis (NTA) NS300 system (Malvern, UK). Cryo-electron microscopy (Cryo-EM) was used for visualization of mEVs. In short, Cryo-EM samples were prepared by applying 3 μL mEV solution on glow discharged 200 mesh gold Quantifoil (2/2) grids (Electron Microscopy Sciences, USA). The grids were vitrified in liquid ethane using a Vitrobot Mark IV (Thermo Fisher, NL). Images were acquired using a TALOS F200C-G2 (Thermo Fisher, NL) operated in low dose mode at 200 kV and equipped with a Falcon 4i detector (Thermo Fisher, NL).

The expressions of EVs common markers were detected by western blotting (WB). In brief, mEVs were washed with PBS and RIPA lysis buffer (Millipore) with a proteinase inhibitor cocktail (Roche diagnostics, cOmplete™) was added. Proteins were separated by electrophoresis on 10 % bisacrylamide gels in 1x electrophoresis buffer. Afterwards the proteins were transferred to a 0.45 μm nitrocellulose (NC) blotting membrane (GE Healthcare) using wet transfer. The blots were blocked with 1x animal-free blocking buffer (Cell Signaling Technologies, 15019) overnight at 4 °C. Thereafter, the blots were washed three times with TBS-Tween® (TBST) for 10 min and then incubated overnight with corresponding antibody: CD81 (Santa Cruz Biotech, sc-166029), Alix (Santa Cruz Biotech, sc-53540), HSP-70 (Santa Cruz Biotech, sc-32239), CD9 (Thermo Fischer, MA1-80307), AnxV (Santa Cruz Biotech, sc-74438) in 2 % bovine serum albumin (BSA) (Sigma Aldrich) dissolved in TBS-T. The blots were treated with enhanced chemiluminescence (ECL) using ECL Prime Western Blotting Detection Reagent Kit (GE Healthcare) to visualize the protein bands with ChemiDoc™MP Imaging System (Bio-Rad) machine and Imagelab 6. software.

To identify EVs surface markers, the EV analyses were performed with Cytek® Amnis® CellStream® Flow Cytometer: To 3 μl mEVs, 3 μl CD81-Alexa488 (1:100, R&D Systems, Inc., Minneapolis, MN, USA), or 3 μl CD9-Alexa647 (1:100, R&D Systems, Inc., Minneapolis, MN, USA) antibodies were added. Thereafter 10 μl PBS was added and samples were incubated overnight at 4 °C. The next day PBS was added to a volume of 150 μL. Small particle setting was used and detection was set up for 60 s with the following settings FSC = 5 and SSC = 5. As negative controls, only antibodies were measured with the same settings. The gating strategy was based on fluorescence channel 488 and 647, and positive events per ml were determined.

### 2.3 Cell culture

Human bone marrow MSCs (hBMSCs, passage 2–5) were maintained in a log phase in a humidified atmosphere with 5 % CO_2_ at 37 °C with cell culture media composed of α-MEM (A14090, Gibco) supplemented with 10 % fetal bovine serum (Gibco) and 1 % penicillin–streptomycin (Gibco). hBMSCs were isolated from bone fragments obtained as surgical excess material following total hip arthroplasty at the Department of Orthopedics (Radboudumc, Nijmegen, the Netherlands) from anonymized patients. In line with the criteria as set by the International Society for Cellular Therapy (ISCT), hBMSCs were immunophenotypically characterized to express MSC markers (>95 % immunopositive for CD90 and CD105, and >85 % immunonegative for CD45).

### 2.4 Osteogenic differentiation *in vitro*

To initial osteogenic differentiation of hBMSC basic osteogenic induction media (OM) that are α-MEM containing 10 % v/v EV-depleted FBS prepared by 18 h UC [56] and 1 % penicillin-streptomycin, as well as 10 mM β-glycerophosphate disodium salt hydrate, 10^−8^ M dexamethasone and 50 μg/ml ascorbic acid were utilized for cell culture. On day 7 of osteogenic differentiation the expression of type I collagen by cells was tested through western blotting. Cells were washed with PBS and RIPA lysis buffer (Millipore) with a proteinase inhibitor cocktail (Roche diagnostics, cOmplete™) was added. Equal proteins were loaded onto 10 % SDS-PAGE and subsequently transferred onto 0.45 μm NC membrane for 120 min at 275 mA. The membrane was blocked with 5 % skim milk overnight at 4 °C. After that the blots were incubated overnight with anti-col1a1 (1:1000, Merck ABT257).

The alkaline phosphatase enzymatic activity (ALP) and the calcium content were tested as previously described [57]. Briefly, 0.5 M alkaline buffer solution (Sigma) and samples (1:5) were incubated with substrate solution (5 mM pNPP disodium salt hexahydrate) (Sigma) for 1 h at 37 °C. The conversion in this assay results in 4-Nitrophenol, and the absorbance was read at 405 nm with Clariostar spectrometer (BMG Labtech). To measure calcium content the cell culture wells were incubated overnight in 0.5 M acetic acid at room temperature. The working solution consisted of 5 % 14.8 M ethanolamine (Merck)/boric acid buffer (BOOM) [pH 11], 5 % Ortho-Cresolphthalein Complexone (Merck), and 2 % hydroxyquinoline (Sigma). Samples mixed with working solution and incubated at RT for 10 min. The absorbance of the plates was measured with Clariostar spectrometer (BMG Labtech) at 570 nm.

To visualize mineralization, histochemical staining of hBMSCs was performed. hBMSCs were washed with PBS and then fixed in 200 μl 70 % ethanol for 10 min. Then the wells were washed one time with demi water. Afterwards the wells were stained with 200 μl 2 % alizarin red s for 10 min. Then the cells were washed with demi water twice and photographed with Nikon D7500.

### 2.5 Human bone organ preparation and culture

Human femoral heads were freshly obtained from the Department of Orthopedics (Radboudumc, Nijmegen, the Netherlands) as surgical excess material following total hip arthroplasty from anonymized patients. Procedures were performed in accordance with the Dutch code of conduct for responsible use of human tissue in medical research. To create standard-sized bone discs, trabecular osteochondral cores were inserted into a custom-made mold with a diameter of 8 mm and a height of 4 mm. The bone discs were secured into a custom-made holder, after which four defects (Ø1.1 mm × 3 mm) were drilled into the bone discs using a drill press (8450468, Bosch PBD40, Germany). mEVs were injected into all defects four times in two weeks. Bone discs were cultured in a 24-well plate within OM and stored in an incubator at 37 °C with 5 % CO_2_.

### 2.6 Fluorochrome labeling

Two fluorochrome labels (Calcein (CO3050, Sigma-Aldrich); Alizarin Red (Alizarin-3-methyliminodiacetic acid, A3882, Sigma-Aldrich) were administered on the 7th and 14th day respectively through OM at a concentration of 25 μg/ml for 24 h [58]. After 24 h of incubation, bone discs were transferred to a clean 24-well plate and washed one time with PBS for 5 min before fresh OM was added. Post-incubation, bone discs were imaged using widefield microscopy (Zeiss Axio Imager 2). To quantify fluorescence intensity along the walls of defects, the donuts labeling centered on the hole have been designed and areas within inner circles have been excluded. Two corresponding and separate channels for Calcein Green and Alizarin Red were utilized in Fiji/ImageJ (National Institutes of Health (NIH), United States).

### 2.7 Binding of mEVs to type I collagen

mEVs were fluorescently labeled using the PKH67 Green Fluorescent Cell Linker Kit kit (Merck). The quantitative binding experiment was performed in 100 μg/ml collagen-coated tissue culture plastic (Advanced Biomatrix® Collagen I, bovine, Cat# 5005). Fluorescently labeled mEVs were added in increasing doses to the wells and incubated for 24 h at 4 °C. The receiving media (unbound part of mEVs) were collected and regarded as ^cb-^mEVs. The wells were washed 3 × with PBS and the fluorescence from ^cb^^+^mEVs was quantitatively measured using a fluorescent plate reader (BioTek), and observed using IVIS (Xenogen VivoVision IVIS Lumina II, PerkinElmer, Waltham, MA, USA).

### 2.8 mEVs labeling and cellular uptake

mEVs were labeled with DiD stain (Invitrogen) according to the manufacturer's instructions. Briefly, EVs were mixed with DiD stain (10 μM) and incubated at room temperature for 15 min in the dark. The labeled EVs were then washed two times with PBS at 100,000 g for 90min at 4 °C. The final EV pellet was suspended in PBS. For uptake assay, 3 × 10^4^ hBMSCs were seeded in each well of collagen-coated tissue culture plastic and incubated with DiD-labeled EVs at 37 °C. After 6 h, cells were washed with PBS and fixed with 4 % paraformaldehyde. The cell nucleuses were stained with DAPI. The imaging was performed using a confocal laser-scanning microscopy (Nikon, Japan).

### 2.9 Proteomic analysis of mEVs and ^cb-^mEVs

Proteins associated with or incorporated in mEVs and ^cb-^mEVs were identified through mass spectrometry by the Radboud Technology Center. Briefly, mEVs (2e+09 particles/each well) were added to collagen-coated tissue culture plastics and incubated for 24 h at 4 °C to collect ^cb-^mEVs. mEVs and ^cb-^mEVs (10 μg in PBS) were precipitated by adding 3 ml ice cold ethanol to 1 ml of sample followed by overnight precipitation at −80 °C and subsequent 15min centrifugation at 14.000*g*. Protein pellets were dissolved in 30 μl 8M urea 10 mM Tris-HCl (pH 8) prior to subsequent reduction and alkylation of cysteine residues by addition of 1 μl 50 mM dithiothreitol and 1 μl 50 mM chloroacetamide and subsequent incubation at room temperature for 30 min in the dark, respectively. Next, 120 μl 50 mM ammoniumbicarbonate (pH 8) and 0.5 μl Trypsin (0.2 μg/μl) were added and samples were incubated overnight at 37 °C. Tryptic digests were analyzed by nanoflow liquid chromatography (Evosep One, Evosep Biosystems) coupled online to a trapped ion mobility spectrometry – quadrupole time-of-flight mass spectrometer (timsTOF Pro2, Bruker Daltonics) via a nanoflow electrospray ionization source (CaptiveSprayer, Bruker Daltonics). Tryptic peptides were separated by C18 reversed phase liquid chromatography (Evosep EV1137 30SPD performance column; 150 mm length x 0.150 mm internal diameter, 1.5 μm C18AQ particles) using the pre-programmed 30 samples per day (30SPD) Evosep One method. The mass spectrometer was operated in positive ionization mode using the default data independent acquisition – Parallel Accumulation SErial Fragmentation (dia-PASEF) [59] instrument method: 0.6–1.6 1/K0 mobility range, 100–1700 *m/z* mass range, 100 ms accumulation time, 100 ms ramp time, 26 Da mass width, 1 Da mass overlap, 32 mass steps per cycle, 0 mobility overlap, 1 mobility window. Acquired spectra were streamed directly to ProteoScape (v2025b, Bruker Daltonics) for protein identification and label-free quantitation against the Uniprot Bovine protein sequence database (downloaded Jan 2024) using the following settings: Spectronaut v19 directDIA+ (Fast) workflow, 0.2 precursor PEP cutoff, 0.01 precursor Q-value cutoff, 0.01 protein Q-value cutoff global, 0.01 protein Q-value cutoff, 0.75 protein PEP cutoff, full tryptic specificity, allowed up to 2 missed cleavages, carbamidomethyl (C) as fixed modification and Oxidation (M) as variable modifications, protein group specific peptides were used for quantitation.

To determine enrichment of protein in ^cb^^+^mEVs, the following formula on EV-protein intensity of single protein in ^cb^^+^mEVs was utilized:

$$\text{EV}-\text{protein}\;\text{intensity}=\frac{1-{\text{PI}}_{\text{cb}-\text{mEVs}}/{\text{PI}}_{\text{mEVs}}}{2\times \left({\text{PN}}_{\text{cb}+\text{mEVs}}/{\text{PN}}_{\text{mEVs}}\right)}$$

Where PI refers to protein intensity and PN refers to particle number. Protein with an EV-protein intensity more than 1 was considered enriched in ^cb^^+^mEVs.

### 2.10 Immunomagnetic separation of mEVs

EVs expressing specific surface markers could be isolated through immunomagnetic separation [60]. Likewise, we incubated mEVs (4e+10 particles) with protein A/G immunomagnetic beads (∼500 μg, MCE) pre-coated 20 μg/ml AnxV antibody (Santa Cruz Biotech, sc-32321) in a roller bench for 1 h at room temperature. According to the manufacturer protocol the beads-unbound mEV were isolated using a DynaMag magnet (Thermo Fisher, USA), washed with 0.2-μm-filtered 0.5 % Tween-20-supplemented PBS four times. We collected the supernatant from the first wash as AnxV depleted mEVs (^AnxV-^mEVs). In addition, the beads-bound AnxV positive mEVs were eluted and collected for NTA measurements and flow cytometry analyses.

### 2.11 Preparation of mEVs-laden hydrogel construct

To prepare EVs-laden hydrogel constructs, acidulated collagen solution was mixed with 10 × PBS, neutralized with 0.023 μL of 1 N sodium hydroxide per μL of collagen to achieve the final concentration of 3 mg/mL using 1 × PBS. Then 30 μL of neutralized collagen was added per well of 96-well plates and allowed to polymerize at 37 °C for 60 min. For EVs encapsulation, DiD-labeled mEVs were incorporated into the collagen solution before polymerization. The distribution of mEVs in hydrogel construct was observed using confocal laser-scanning microscopy. Moreover, the *in-vitro* release kinetic of mEVs was assessed by NTA. Briefly, mEVs-laden hydrogel construct was incubated in sterile PBS at 37 °C. On day 1, 3, 6, and 10, the receiving media was collected and replaced by an equal volume of fresh PBS. On day 14, 200 U/ml collagenase D (Roche) was added for digestion of hydrogel after incubation at 37 °C incubator for 1h. The percentage of mEVs released was calculated from the initial quantity of EVs added prior to gelation. For hBMSCs mineralization in 3D collagen-based hydrogel, 2 × 10^4^ hBMSCs were mixed either hydrogel only (Ctrl-col) or containing mEVs (mEVs-col), ^cb-^mEVs (^cb-^mEVs-col) and ^AnxV-^mEVs (^AnxV-^mEVs-col) and cultured within OM medium for 14 days.

### 2.12 Evaluation of bone repair within mEVs, ^cb-^mEVs or ^AnxV-^mEVs-laden hydrogel construct *in vivo*

All animal care and related experimental procedures were carried out according to the guidelines of Chinese Research Council's and all procedures were approved by the ethical committee for animal care and use of Shenzhen University (SYXK 2022-0302). To evaluate the bone repair effect of three types of mEVs-laden constructs *in vivo*, we created the femoral condyle defect model in OVX-induced osteoporotic SD rats (females; 320–380g, 16 weeks old) according to the previously described method [61,62].

*Surgical procedure* Firstly, bilateral ovariectomies were performed to induce osteoporosis under general anesthesia in SD rats (females; 200–250g, 12 weeks old). After four weeks the bilateral femoral condyle defects operation was conducted in all rats. Each animal was anesthetized and immobilized in a supine position. A longitudinal incision was made along the direction of the sartorius muscle, and the distal femur was exposed after opening the skin and separating the muscle. After exposure of distal femur, the knee was flexed, and the soft tissues including muscle and ligament were moved aside to let the femoral condyles completely expose. A defect (2.4 mm in diameter, 3 mm in depth) was created between two femoral condyles. After the creation of the femoral condyle defect, the defected holes were covered by implanting either ctrl-col or containing 9e+07 particles of mEVs-col, ^cb-^mEVs-col and ^AnxV-^mEVs-col. All rats were housed and maintained in Specific Pathogen Free facility.

*Micro-CT scanning* Four weeks post operation, all rats were euthanized to excise the femur for fixation in neutral buffered formalin for 36 h. To assess the bone repair at defect sites, the collected samples were scanned by high-resolution micro-computed tomography (μCT) system (Quantum FX, Perkin-Elmer); The scanning parameters were as follows: 70 kV, 100 μA, 144 μm. The μCT analyser software (Analyze 14.0, Mayo, USA) was used to define a region of interest (ROI) based on the two-dimensional (2D) images which representing the defects surrounding newly formed bone tissue to obtain a volume of interest (VOI) dataset. Once the views around the ROI were reconstructed, bone volume/total volume (BV/TV) was measured.

*Histological analysis* Formalin-fixed femoral condyles were decalcified in 10 % ethylenediaminetetraacetic acid (EDTA) solution followed by embedding in paraffin for 4 weeks. The tissues were then sectioned into 7 μm thick slices. The sectioned tissues were placed on slides and dried overnight for subsequent histological analysis, including hematoxylin-eosin (HE) and Verhoeff's Van Gieson (EVG) staining as previous study [61]. Sections were further scanned with a NanoZoomer Digital Pathology System (Hamamatsu, Bridgewater, USA).

### 2.13 Statistical analysis

Results were analyzed in GraphPad Prism 9 using unpaired two-tailed Student's t tests with two groups and one-way ANOVA with a Dunnett post hoc test with more than two groups as per specific *ex vivo* experiment. Data for animal experiments were evaluated using paired two-tailed Student's t tests within each group (Ctrl-col & mEVs-col; Ctrl-col & ^cb-^mEVs-col; Ctrl-col & ^AnxV-^mEVs-col). A p-value below 0.05 (p < 0.05) was considered to be statistically significant.

## 3 Results

### 3.1 Isolation and characterization of mEVs

**Fig. 1:**
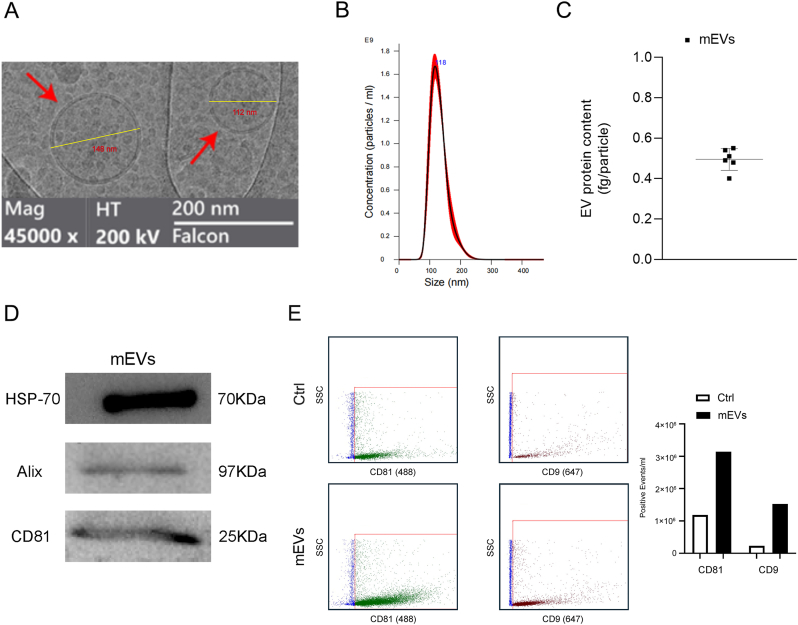
Isolation and characterization of mEVs. A. Morphological appearance of mEVs upon electron microscopy (Cryo-EM). B. Size distribution of mEVs (n = 3). C. Determination of mEV protein content (n = 6). D. Western blotting analysis of protein levels of HSP-70, Alix, and CD81 in mEV lysates. E. Flowcytometry measurement of mEVs expressing CD81 (fluorescence channel 488 nm) or CD9 (fluorescence channel 647 nm). Ctrl: corresponding antibody in pbs. SSC: side scatter. Data were expressed as means ± standard deviation (SD).

Cryo-EM showed that mEVs were relatively regular in morphology with a circular shape, surrounded by a well-defined lipid bilayer membrane with diameters <200 nm (Fig. 1A), having a yield of 5e+10 particles/ml and a median particle size of 118 nm as determined by NTA (Fig. 1B). The EV protein content used as an indication of EV purity [63] was 0.5 ± 0.05 fg/particle (Fig. 1C). WB results further confirmed that mEVs expressed the common EV markers HSP-70, Alix, and CD81 (Fig. 1D). Of note, CD81 or CD9 positive mEVs were also identified with flow cytometry (Fig. 1E). Taken together, these results proved the successful extraction of mEVs from cow milk.

### 3.2 mEVs stimulate osteogenesis in cell and organ cultures

**Fig. 2:**
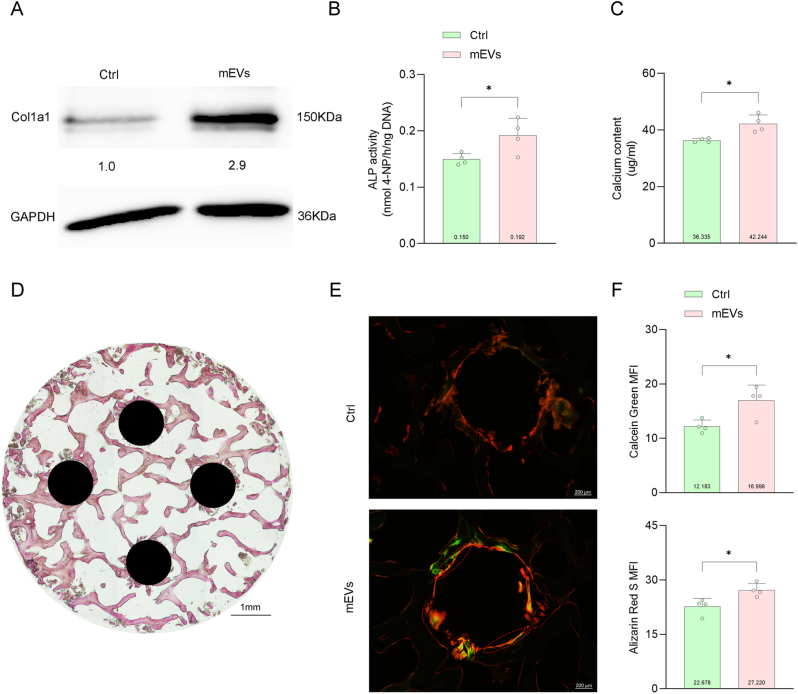
mEVs stimulate osteogenesis in cell and organ cultures. A. Western blotting for Col1a1 protein expression at day 7. B. Quantification of ALP activity at day 14 and C) Calcium content of hBMSCs cultured in OM (Ctrl) or OM within mEVs at day 21 (n = 4). D. Methylene blue/basic fuchsine staining of the bone disc within four drilled holes (indicated by black circles). E. Representative images of the drilled holes stained with calcein green on the 7th day and alizarin red s on the 14th day for ctrl (pbs) and mEVs injection. F. Mean fluorescence intensity (MFI) of calcein green and alizarin red s on inner edges of defects (dots represent holes of each sample). Scale bars correspond to 1 mm in 2D and 200 μm in 2E. Data were expressed as means ± standard deviation (SD). ∗p < 0.05. (For interpretation of the references to colour in this figure legend, the reader is referred to the Web version of this article.)

To clearly assess whether bulk SEC-purified mEVs have a pro-osteogenic effect on MSCs, we exposed hBMSCs to basic OM with or without supplementation with mEVs (5e+06 particles/ml). EV supplementation resulted in an enhanced expression of type I collagen at day 7 (Fig. 2A) as determined by WB, increased activity of ALP at day 14 (Fig. 2B), and a higher calcium deposition at day 21 (Fig. 2C). Furthermore, a human bone *ex vivo* organ culture model was established, in which hBMSCs and osteoblasts are preserved in their native three-dimensional environment [64]. After 14 days of culture in OM, methylene blue/basic fuchsine staining showed preservation of trabecular bone structures with viable osteocytes. To test the effect of local mEV delivery on repair of a bone defect, 4 holes were drilled into the bone explant directly after preparation (Fig. 2D). mEV injection into these holes of the bone explants showed to increase fluorochrome uptake along the walls of these holes after day 14 of culture (Fig. 2E and F). Collectively, these results imply that mEVs stimulate osteogenic differentiation of hBMSCs *in vitro* and bone formation *ex vivo.*

### 3.3 Binding to collagen separates mEVs with osteogenic functionality in cell cultures

**Fig. 3:**
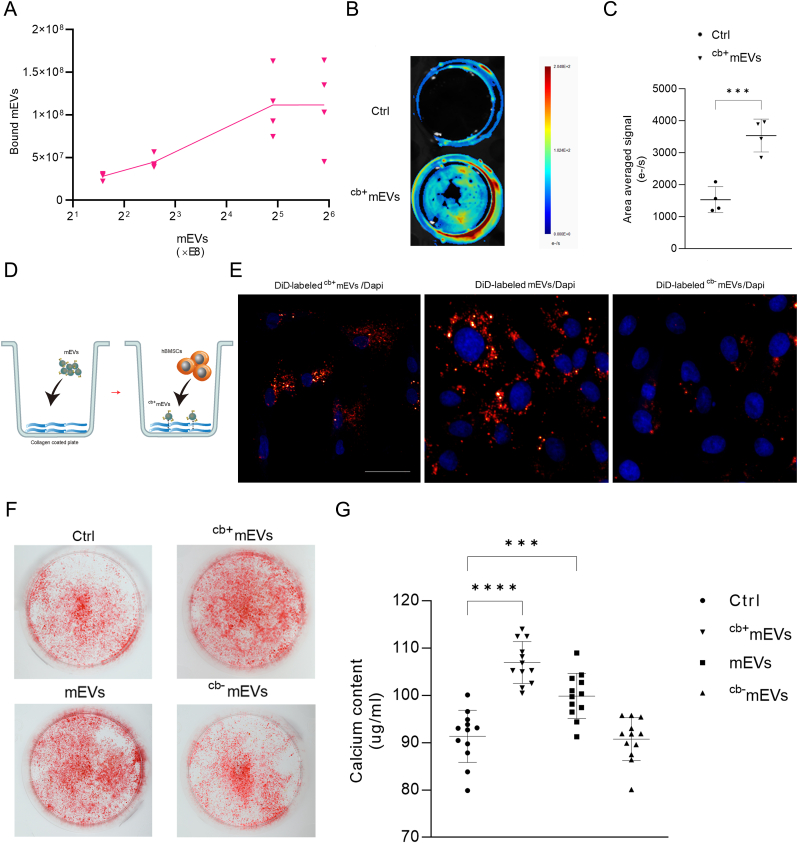
Binding to collagen separates mEVs with osteogenic functionality in cell cultures. A. Quantification of mEV with collagen binding properties (n = 4). B. Representative IVIS image showing fluorescently labeled ^cb^^+^mEVs bound to collagen-coated tissue culture plastic (Ctrl: label agent in pbs). (C) Quantitative area averaged signal in from fluorescently labeled ^cb^^+^mEVs bound to collagen-coated tissue culture plastic (Ctrl: label agent in pbs) (n = 4). (D) Schematic representation showing sequential mEVs loading and hBMSCs seeding on collagen-coated tissue culture plastic. (E) Uptake of ^cb^^+^mEVs, mEVs, ^cb-^mEVs by hBMSCs within 6h. (F) Representative alizarin red staining of hBMSCs cultured on collagen-coated tissue culture plastic (PBS control, ^cb^^+^mEVs, mEVs or ^cb-^mEVs) at day 21. (G) Calcium content of hBMSCs cultured on collagen-coated tissue culture plastic (PBS control, ^cb^^+^mEVs, mEVs or ^cb-^mEVs) at day 21 (n = 12). Data were expressed as means ± standard deviation (SD). ∗∗∗p < 0.001; ∗∗∗∗p < 0.0001. Scale bars correspond to 100 μm in 3E. (For interpretation of the references to colour in this figure legend, the reader is referred to the Web version of this article.)

Using a collagen-binding experiment for mEVs with collagen-coated tissue culture plastic, the binding curve revealed that 4 % of the input particles were able to bind to collagen, and saturation occurred from mEVs numbers of 3e+09 particles (Fig. 3A). Moreover, fluorescently labeled ^cb^^+^mEVs were observed and the signal intensities were calculated using IVIS optical imaging (Fig. 3B and C). To evaluate the effect of ^cb^^+^mEVs on osteogenic differentiation of hBMSCs, cells were seeded on the collagen-coated plate with or without ^cb^^+^mEVs (Fig. 3D). Within 6h (from cell seeding to fixation), we observed that more than 60 % of hBMSCs specifically took up ^cb^^+^mEVs (Fig. S1: Fluorescence image of negative control). Both mEVs and ^cb-^mEVs were internalized by adherent cells within 6 h. However, noticeably fewer labeled ^cb-^mEVs were observed in the perinuclear region compared to mEVs (Fig. 3E). By Cryo-EM and NTA analysis we confirmed that ^cb-^mEVs are true EVs of similar size and purity as bulk mEVs (Fig. S2). More importantly, alizarin red s staining showed significantly more mineralization after 21 days of OM culture by hBMSCs cultured on collagen-coated plates with ^cb^^+^mEVs and mEVs, and less mineralization was observed with ^cb-^mEVs stimulation (Fig. 3F). Consistently, the calcium content of hBMSCs under control (PBS) conditions was 91.4 ± 5.5 μg/ml while ^cb^^+^mEVs presence resulted in 106.9 ± 4.4 μg/ml and 99.9 ± 4.8 μg/ml with mEVs stimulation but 90.8 ± 4.6 μg/ml upon ^cb-^mEVs stimulation, respectively (Fig. 3G). Taken together, these data show that ^cb^^+^mEVs are the functional subpopulation of mEVs that drives osteogenic differentiation of hBMSCs.

### 3.4 AnxV mediates the osteogenic effect of ^cb^^+^mEVs

**Fig. 4:**
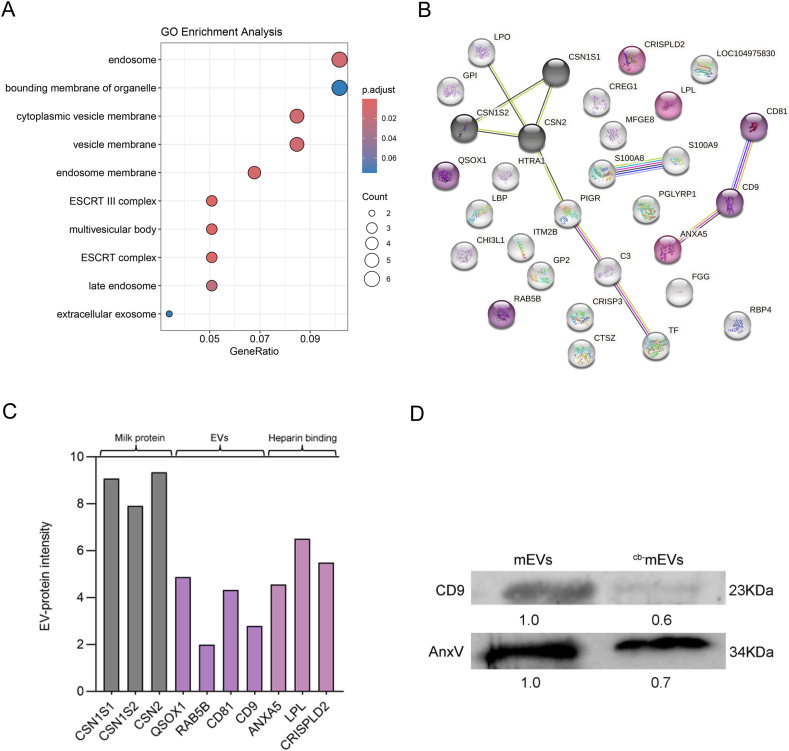
AnxV was enriched in CD9 positive ^cb^^+^mEVs. A. The classification of proteins enriched in ^cb^^+^mEVs according to the cellular components. B. The STRING analysis of the proteins in extracellular region for protein-protein interactions. C. The EV-protein intensity of proteins (extracellular components) in ^cb^^+^mEVs. D. Western blotting analysis of protein levels of CD9, AnxV in mEVs and ^cb-^mEVs lysates.

As we cannot isolate sufficient quantity of the ^cb^^+^mEV subpopulation for proteomic analysis, we performed proteomic profiling of (bulk) mEVs and ^cb-^mEVs to investigate proteins enriched in ^cb^**^+^**mEVs that facilitate collagen binding. The comprehensive mass spectrometric analysis revealed that mEVs possess over 260 quantifiable proteins. Given that ^cb^^+^mEVs account for 4 % of the bulk mEVs, the EV-protein intensity of 73 proteins is greater than 1, which were selected for further analysis (Table S1). The GO term “cellular components” showed that all 73 selected proteins were main EV-associated proteins (Fig. 4A), indicating the specific binding between type I collagen and EVs. Next, proteins located in the extracellular region and their interactions were mapped (Fig. 4B). One single interaction chain was identified, comprising CD9, CD81 and AnxV that was digitally stained as heparin-binding protein. Three casein-related proteins have been labeled as milk protein. The EV-protein intensity of the extracellular proteins exceeded 1.90 (Fig. 4C). Since CD9 has been reported as one specific marker among tetraspanins present on some EV subpopulations [65,66], we speculated AnxV was enriched in CD9 positive ^cb^^+^mEVs. WB results showed that compared to (bulk) mEVs the protein levels for CD9 and AnxV decreased 40 % and 30 % in ^cb-^mEVs (Fig. 4D).

**Fig. 5:**
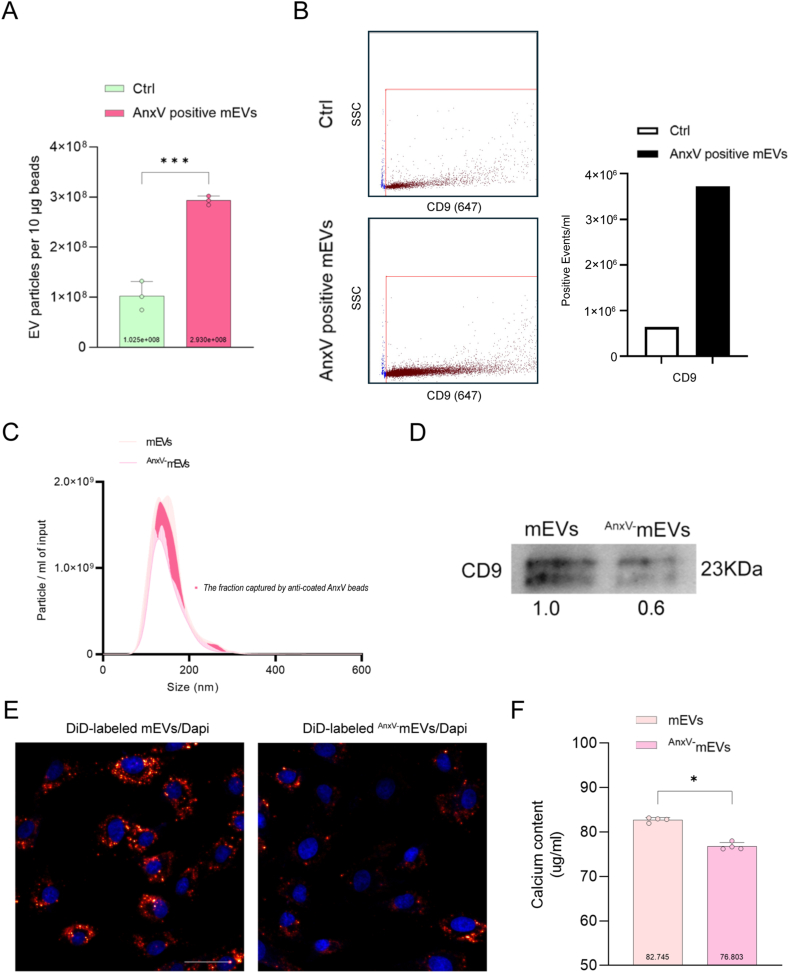
Depleting AnxV diminished mEV-induced hBMSCs osteogenesis. A. Number of AnxV positive mEVs (n = 3). Ctrl: beads-AnxV antibody-pbs complex. B. Flowcytometry measurement of AnxV positive mEVs expressing CD9. Ctrl: corresponding antibody in pbs. C. NTA of mEVs before and after incubation (inc.) with magnetic beads coated with anti-AnxV antibody (n = 2). D. Western blotting analysis of protein levels of CD9 in mEVs and ^AnxV-^mEVs lysates. E. The uptake of mEVs or ^AnxV-^mEVs by adherent hBMSCs within 6h. F. Calcium content of hBMSCs cultured in OM within mEVs or ^Anxv-^mEVs (n = 4). Data were expressed as means ± standard deviation (SD). ∗p < 0.05, ∗∗∗p < 0.001. Scale bars correspond to 100 μm in 5E.

Thereafter, to assess the role of AnxV on mEVs-induced osteogenesis, immunomagnetic beads separation was conducted to collect ^AnxV^^+^mEVs and ^AnxV-^mEVs. The particle amount of ^AnxV^^+^mEVs was 1.9e+08 particles per 10 μg beads (Fig. 5A). Flow cytometry analysis confirmed the presence of CD9 on ^AnxV^^+^mEVs (Fig. 5B). In addition, NTA analysis showed the shifted size distribution of mEVs due to the removal of the fraction captured by anti-coated AnxV beads (Fig. 5C). WB revealed that the protein levels for CD9 decreased 40 % in ^AnxV-^mEVs compared to mEVs (Fig. 5D). More importantly, we observed that hBMSCs took up much fewer ^AnxV-^mEVs (Fig. 5E). Calcium deposition in hBMSCs was significantly reduced after 21 days of OM culture with ^AnxV-^mEVs stimulation compared to mEVs (Fig. 5F). In addition, to investigate the role of AnxV on mEVs binding to collagen, we checked the binding capacity of ^AnxV-^mEVs in collagen-coated plates. IVIS imaging showed less signal of labeled collagen-binding ^AnxV-^mEVs (^cb+ AnxV-^mEVs) compared to that of ^cb^^+^mEVs. Decreased calcium deposition by hBMSCs was determined in coated collagen plate within ^cb+ AnxV-^mEVs (Fig. S3). Collectively, these data strongly suggest that AnxV is enriched on ^cb^^+^mEVs and contributes to mEV-induced osteogenesis.

### 3.5 ^cb^^+^mEVs in 3D hydrogel constructs enhance hBMSCs mineralization

**Fig. 6:**
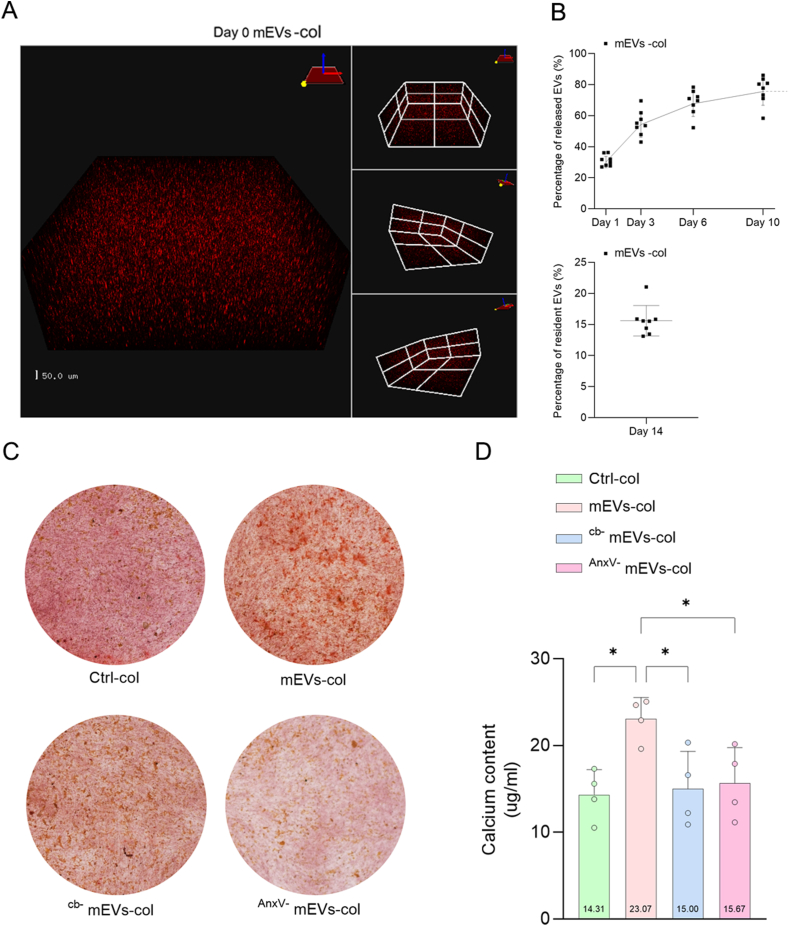
^cb^^+^mEVs in 3D hydrogel construct enhanced hBMSCs mineralization. A. Laser scanning-confocal microscope showing fluorescently labeled mEVs in 3D collagen hydrogel at day 0. B. Graphical representation of 3D encapsulated fluorescently labeled mEVs release from collagen-based hydrogels over time (n = 8). C. Representative alizarin red staining of hBMSCs and D) Calcium content of hBMSCs encapsulated in 3D collagen-based hydrogel containing PBS (Ctrl) or mEVs or ^cb-^mEVs or ^AnxV-^mEVs for 21 day OM culture (n = 4). Data were expressed as means ± standard deviation (SD). ∗p < 0.05. Scale bars correspond to 50 μm in 6A. (For interpretation of the references to colour in this figure legend, the reader is referred to the Web version of this article.)

Next, we strived to apply mEV subpopulations therapeutically to heal bone defects. For this, we engineered hydrogel constructs with encapsulated mEVs and determined the EV release kinetics for 14 days in PBS at 37 °C. Fluorescently labeled mEVs were observed in 3D hydrogel constructs at day 0 using laser confocal microscopy (Fig. 6A). Robust release of mEVs was observed from day 1 to day 6, during which 68.1 ± 8.7 % mEVs was released from the hydrogel construct. However, thereafter the release plateaued and between day 6 up to day 10 only 7.8 % ± 1.2 mEVs was released. Because the particle amount of mEVs in receiving PBS on day 14 was undetectable with NTA, we degraded the hydrogel to measure the particle amount of still encapsulated mEVs and found 15.6 % ± 2.5 mEVs to be still entrapped in the hydrogel (Fig. 6B). Next, we mixed hBMSCs into the EVs containing hydrogel constructs and after 14 days of culture in OM medium more calcium staining was apparent from Alizarin red S staining for mEVs-col compared to ^cb-^mEVs-col and ^AnxV-^mEVs-col (Fig. 6C). The highest calcium content by hBMSCs was found for mEVs-col (Fig. 6D). Consequently, these data demonstrate that mEVs were successfully encapsulated in 3D hydrogel constructs and we deduced from these data that in particular the ^cb^^+^mEVs population is responsible for the enhanced hBMSCs mineralization *ex vivo*.

### 3.6 Removal of ^cb^^+^mEVs eliminates the effect of mEVs-mediate acceleration of osteoporotic bone regeneration

**Fig. 7:**
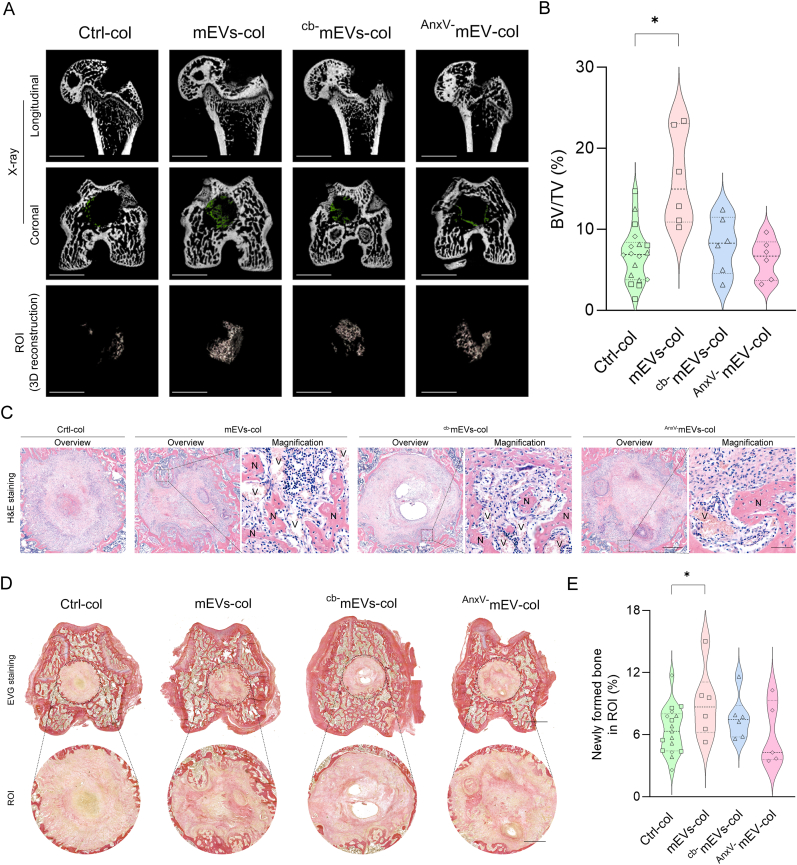
Removal of ^cb^^+^mEVs eliminates the effect of mEVs-mediated acceleration of osteoporotic bone regeneration. A. Representative 2D micro-CT X-ray images from longitudinal and coronal view (The newly formed bone tissues within the defects were highlighted by green) and 3D reconstructions of ROI in femoral condyle defects. B. Bone morphometry analysis at 4 weeks of bone defects treated with ctrl-col, mEVs-col, ^cb-^mEVs-col, or ^AnxV-^mEVs-col (n = 6 for each comparison). C. Representative images of H&E staining of the femoral condyle defects with mEVs-col, ^cb-^mEVs and ^AnxV-^mEVs in collagen, or collagen only (ctrl). N: newly formed bone, V: micro-vessels. D. Representative images of EVG staining of the femoral condyle defects mEVs-col, ^cb-^mEVs and ^AnxV-^mEVs in collagen hydrogel, or collagen hydrogel only (ctrl). Semi-quantitative analysis of (E) the area fractions of newly formed bone by staining intensity in separate channels through ImageJ software (n = 6 for mEVs-col and ^cb-^mEVs-col groups comparison and n = 5 for ^AnxV-^mEVs-col group). “□” refers to the rats received mEVs-col and Ctrl-col treatment; “△” refers to the rats received ^cb-^mEVs -col and Ctrl-col treatment; “◇” refers to the rats received ^AnxV-^mEVs-col and Ctrl-col treatment. ∗p < 0.05. Scale bars correspond to 2.5 mm in 7A. In histology analysis, scale bar = 500 μm for overviewed image and 50 μm for magnified image in 7C; scale bar = 1 mm for overviewed EVG staining image and 500 μm for ROI image in 7D. (For interpretation of the references to colour in this figure legend, the reader is referred to the Web version of this article.)

To further evaluate mEV and ^cb^^+^mEVs efficacies toward bone regeneration, we implanted the mEVs, ^cb-^mEVs and ^AnxV-^mEVs laden-hydrogel constructs into femoral condyle defects of OVX-induced osteoporotic rats. 2D micro-CT X-ray images revealed massive bone loss in the distal femoral diaphysis from longitudinal view, confirming OVX-induced osteoporotic bone conditions following ovariectomy. New bone formation occurred in all experimental groups but was the most pronounced for bone defects filled with mEVs-laden hydrogels (Fig. 7A). Bone morphometry analysis showed that only the mEVs-col group exhibited a 2.4-fold increase in bone tissue volume/total tissue volume (BV/TV%) compared to collagen controls, while groups lacking ^cb^^+^mEVs showed no improvement for this parameter (Fig. 7B). Thereafter, we performed a histological analysis to investigate the ossification within the ROI. The images within closed defects surrounded by mature bone are the eligible ones for ROI determination. The inclusion of samples was based on careful selection of properly created bone defects (Table S2). H&E staining showed that new blood vessels identified by yellow cell nuclei in luminal structures around the newly formed bone were present in the ROI of all included samples slides (Fig. 7C). To distinguish between connective tissue (yellow), newly formed bone (pink) and host bone tissue (bright red), we carried out EVG staining (Fig. 7D). Moreover, we quantified the amount of newly formed bone within the ROI using consecutive sections. The area fractions of newly formed bone in the defects implanted with mEVs-col exhibited a 1.4-fold increase compared to collagen controls (Fig. 7E), while removal of ^cb^^+^mEVs abolished the effect of mEVs on bone formation. Collectively, these data support the contribution of the ^cb^^+^mEVs subpopulation to mEV-mediated acceleration of orthotopic osteoporotic bone defect regeneration.

## 4 Discussion

Most research on EVs focuses on their use in drug delivery, regenerative medicine, and the identification of EV-based biomarkers [67,68], where EVs have been regarded as a single, homogeneous population. However, emerging evidence has emphasized the presence of distinct EV subpopulations [69,70]. This study builds on these findings by highlighting the functional heterogeneity of mEVs, which can be explored after separating mEV subpopulations based on their collagen-binding property. This supports our hypothesis that ECM-binding capacity is an “EV-inherent feature” associated with functionality toward bone regeneration.

Emerging evidence shows that non-EV particles and proteins independent of EVs are susceptible to being co-isolated during ultracentrifugation (UC), leading to sample contamination [71]. Introducing SEC purification following ultracentrifugation, we obtained mEVs with higher purity expressing CD9 and CD81. These UC/SEC isolated mEVs were shown to stimulate mineral apposition of *ex vivo* bone and kept the positive effect on BMSC commitment toward an osteogenic phenotype without supplementing additional factors such as Ca^2+^ or bone morphogenetic protein 2 (BMP2) [[72], [73], [74]]. Attributing functional performance to a specific EV size fraction in downstream assessments is challenging [70]. It has been demonstrated that CD9 positive EVs mainly are present in the size range 50–100 nm and CD81 in 50–200 nm [70], meaning during SEC isolation the 70 nm resin pore size inevitably resulted in a large loss of CD9 and CD81 positive mEVs smaller than 70 nm. In the present study, the vast majority of mEVs are within the range of 100–200 nm according to cryo-EM imaging and NTA analysis. Therefore, to determine functional subpopulations within bulk mEVs, we turned to their surface moieties [49,75].

Specific molecules present on the surface of EVs are considered to facilitate the EV-ECM interactions through covalent or hydrogen bonding [47]. Typically, to increase EVs retention within hydrogels for eliciting specific biological response, selective molecular interactions are desirable. A very recent paper demonstrated that EVs’ immobilization in the type I collagen hydrogel was mediated by surface proteins RGD-binding integrins in a composition-dependent manner [76]. Furthermore, this specificity on surface biomolecules shapes EVs heterogeneity. For instance, *Lai* et al. leveraged specific lipid-binding ligands such as cholera toxin B and shiga toxin B to selectively isolate different MSC-EV subtypes with different protein and RNA contents. Notably, only shiga toxin B-binding EVs were found to express fibronectin with extra domain that are associated with promoting recruitment and adhesion of reparative cells [77]. In our study, we made use of the binding between mEVs and type I collagen to separate subpopulations, and a constant proportion of input mEVs binding to the collagen was calculated. The complete loss of osteogenic functionality in mEVs after the removal of ^cb^^+^mEVs implied that ^cb^^+^mEVs have been efficiently separated from the bulk mEVs. In addition, it has been reported that EVs still can be taken up by surrounding cells such as human endothelial colony-forming cells in the collagen-bound condition [54]. Herein, labeled ^cb^^+^mEVs accumulated massively around the nuclei of hBMSCs during culture on collagen plates and influenced the osteogenic differentiation of hBMSCs. This suggests that ^cb^^+^mEVs dynamically detached from collagen and entered into the cells.

Through bioinformatics analysis, we deduced that AnxV is associated with CD9 and CD81 in the ^cb^^+^mEV subpopulation. This is interesting as one recent paper reported that AnxV derived from MVs plays a central role in mediating MV adhesion to collagen and bone mineralization [78]. Specifically, MVs have been isolated from osteoblasts of mice in normal or osteoporotic conditions. In their cases, the inter-subpopulation heterogeneity (compositional and functional differences amongst EVs from various sources) contributed to distinguishing two types of EVs with massive difference in amount of AnxV. Herein the subsets of mEVs stemmed from the same bulk, as called intra-subpopulation heterogeneity (differences in EVs within subpopulations derived from the same source), have been identified and isolated through immunomagnetic separation. However, the cellular uptake experimental data from the two studies indicated that the reduction of AnxV affects the receptor cells' uptake of EVs. AnxV, widely recognized as an EV marker [79], has been reported to be present in cow milk and capable of binding to collagen [80]. Calcium binding AnxV proteins are essential in mediating proteoliposome attachment to collagen fibrils [81]. The 2D coated type I collagen has negatively charged regions, including specific sites that resemble heparin structures. Therefore, the heparin-binding domain of AnxV could interact with these negatively charged regions on collagen. Moreover, the binding specificity was further enhanced with stabilization by calcium ions [82]. During OM culture, BMSCs can expose PS on their cell membrane facilitating interaction with annexin proteins and calcium-binding proteins like S100A9. These interactions enhance MV uptake by BMSCs and promote mineralization [83]. In our study, the inefficient cellular uptake observed in ^AnxV-^mEVs suggests that PS on BMSCs plays a role. Impaired uptake leads to a reduction in calcium mineralization for hBMSCs, indicating that the binding and potential uptake of mEVs are essential for functionality, and this process depends on AnxV on mEVs.

It is well established that integrin-mediated adhesion to collagen promotes stromal cell osteogenic differentiation [84]. This interaction has been utilized to immobilize EVs within collagen hydrogels for various clinical applications [85]. *Hao* et al. showed that functionalizing a material surface with the integrin α4β1 ligand, LLP2A, improved the binding of MSC-derived EVs and enhanced vascularization [86]. However, the proteomic analysis in the present study did not detect any integrin protein. We speculated that most of the mEVs are small EVs (exosomes), and integrin markers such as integrin β1, are not present on the surface of exosomes [87,88]. Additionally, recent work reported that milk fat globule-EGF factor 8 (MFGE8) was identified as a principal protein in the corona around EVs released by human pluripotent stromal cells (hPSCs) that helps facilitate the uptake of EVs by recipient cells, a process that is crucial for maintaining the self-renewal and pluripotency of hPSCs [89]. In our study, MFGE8 as a protein found in milk, has been deduced as one of extracellular proteins enriched in ^cb^^+^mEVs. Previous paper reported that MFGE8 bound to type I collagen, resulting in promotion of collagen uptake by macrophages and decrease in severity of tissue fibrosis [90]. Therefore, we speculated this contributes to the mEVs binding to the collagen but might alleviate mEVs-induced osteogenesis of hBMSCs.

Collagen hydrogel is a highly biocompatible material, providing a suitable environment for cell adhesion and proliferation. Previously, we demonstrated that osteoclasts and osteoclast-derived EVs encapsulated in collagen hydrogel promote bone regeneration using a mouse tibial bone defect model [91]. Considering the large fraction of mEVs released from collagen hydrogel over 14 days *in vitro*, we here collected the rat femoral condyles in the early phase of bone healing (4 weeks). Osteoporotic bone conditions serve as a challenging scenario for bone healing because it is characterized by decreased bone density, disrupted microarchitecture, and imbalance of bone turnover. These factors collectively weaken the regenerative capacity of bone and delay effective repair [92]. In these conditions, EVs could bring about a more obvious effect on bone regeneration [93]. Therefore, we tested the effect of mEV-laden collagen hydrogels on bone defect repair in an OVX-induced osteoporotic rat bone defect model. To minimize OVX-related variability, each animal received both an experimental and a control treatment. Of note, we observed the presence of microvascular networks in all the samples, which provide essential nutrients, and growth factors to the regenerating bone tissue. EVG staining was conducted to quantify the area fractions of newly formed bone [61]. It showed the collagen deposition appeared from various positions of the defect edges and had the tendency to spread outward. We observed the ^cb-^mEV or ^AnxV-^mEVs groups showed comparable amount of pink, randomly oriented collagen fiber compared to the control group. However, a noticeable increase in fiber density was found in defects filled with hydrogel constructs containing mEVs, suggesting that it is ^cb^^+^mEV subpopulation stimulates mEV-mediated bone formation *in vivo* at 4 weeks after implantation.

A notable innovation of this study is utilizing the specific biomolecular interaction between mEVs and type I collagen for EV subpopulation selection. The foundation for utilizing this inherent property is the high purity of mEVs. EVs isolated from body liquids comprise more lipoprotein and protein contaminants compared to EVs isolated from cell culture, typically MSCs [94]. Therefore, SEC isolation is an essential procedure for purifying mEVs in the present study. While for MSCs-EVs, EVs come from FBS are the main contamination problem. To obtain homogenous functional MSCs-EVs using PEG EVdepleted FBS [95], in combination of further collagen-binding selection could be a desirable method. It has been reported that MSCs-EVs contain the collagen-binding property. In consistent with the binding experiment results of our study, *Huang* et al. demonstrated a constant proportion of bulk MSCs-EVs bound to type I collagen [96]. However, their further research direction turned to control EVs release through introducing mimetic peptides within hydrogel construct. Controlling EVs release by functionalizing the biomaterials has been the focus of investigation, especially in bone regeneration. *Sophie Cox* et al. reported that pre-osteoblast derived-EVs had various release kinetics in different ratio of chitosan-collagen, and 65 %/35 % chitosan-collagen has been utilized as the optimal construct for EVs delivery [80]. However, the released data on EVs in 100 % collagen in their study showed CD63 positive EVs were barely detected in the receiving media, implying CD63 positive EVs have the optimal collagen binding property. Moreover, in the contactless transwell system, the most calcium depositions were observed the 100 % chitosan containing EVs group, suggesting the CD63 positive EVs had the best osteogenic functionality. Recently, enhancing the collagen-binding property by conjugating some artificial peptides has been explored, which contributes to the retention of EVs at target sites [54,97]. However, it seems that these studies overlook the heterogeneity of EVs. Although this approach aids in the retention of EVs, it might not be the bulk effect of EVs that alleviates the inflammatory response, promotes angiogenesis and osteogenesis, but rather the binding peptide that contributes to these effects. In the current study, we focused on native EVs and did not introduce external factors to explore a subset of EVs with collagen-binding properties that can promote osteogenesis.

It is a challenge that EV enrichment technologies have not kept pace with characterization tools in addressing the heterogeneity of EVs [70]. EVs could be characterized at the single-vesicle level but could not be easily enriched. This makes EV subpopulation recovery more difficult in the context of therapeutic applications, where large numbers of EVs are often required [39]. This limited our study to isolate ^cb^^+^mEVs and forced us to indirectly investigate the protein composition in ^cb^^+^mEVs by comparing mEVs and ^cb-^mEVs and use only the bulk and negative functional mEVs parts *in vivo*. However, the images of ^cb-^mEVs uptake by cells implied the presence of ^cb-^mEVs in mEVs may not compete with ^cb^^+^mEVs to enter the recipient cells and negatively influence the effect of mEVs in driving osteogenesis. Therefore, it is imperative to evaluate whether ^cb^^+^mEVs could truly perform better compared to the bulk mEVs in boosting bone regeneration. Future research should explore ways to enrich collagen-binding mEVs. For example, 3D collagen-coated microspheres can be used to increase the binding area for ^cb^^+^mEVs. However, it is not recommended to load mEVs into the microspheres, as the physical properties of EVs and ECM might affect their binding. Furthermore, by exploring the biomolecular interactions between EVs and ECM, we can establish a standard procedure, such as collagen binding (domain) chromatography, to isolate collagen-binding mEVs. Nevertheless, column EV desorption using high salt or acid may harm the integrity of EVs. Knowing which binding protein mediates the collagen attachment or using a unique marker of this EV subpopulation remains essential for further high yield immunoaffinity purification.

The clinical translational potential of ^cb^^+^mEVs is significantly enhanced by overcoming the challenge of heterogeneity common in broader EV populations. Unlike bulk mEVs, which vary in cargo and function, ^cb^^+^mEVs represent a standardized subset, ensuring consistent and predictable therapeutic outcomes. This consistency allows for more reliable clinical applications, particularly in bone repair, compared to EV-functionalized biomaterials, where variability in EV quality compromises results. Compared to traditional materials like autografts and allografts, ^cb^^+^mEVs offer a non-invasive, cell-free alternative, eliminating risks of immune rejection and donor site morbidity, while also providing better integration into the bone matrix due to their collagen-binding properties. Additionally, ^cb^^+^mEVs are more effective than synthetic bone substitutes or growth factor therapies, as they deliver regenerative factors more precisely, promoting localized bone regeneration with fewer side effects. Thus, with their resolved heterogeneity, ^cb^^+^mEVs offer a safe, scalable, and cost-effective solution for bone repair, positioning them as a promising alternative to both traditional and emerging bone repair materials.

## 5 Conclusion

We demonstrate the osteogenic functionality of UC/SEC bulk mEVs. More importantly, focusing on mEV heterogeneity, we harnessed collagen binding to separate ^cb^^+^mEVs and ^cb-^mEVs. Using *in vitro* osteogenic differentiation, *ex vivo* osteogenesis, and *in vivo* osteoporotic bone defect regeneration, we show that the ^cb^^+^mEV subpopulation is responsible for the osteogenic functionality of mEVs but ^cb-^mEVs, that represent the largest proportion of bulk mEVs, have no bone-promoting capacity. AnxV was found to be enriched in the ^cb^^+^mEV subpopulation and essential for mEVs collagen binding and mEVs-mediated bone regeneration.

## CRediT authorship contribution statement

**Peng Wang:** Writing – review & editing, Writing – original draft, Visualization, Validation, Methodology, Investigation, Formal analysis, Data curation, Conceptualization. **Yang Zhang:** Writing – review & editing, Supervision, Resources, Methodology. **Onno J. Arntz:** Methodology, Investigation, Data curation. **Marina C. Oliveira:** Writing – review & editing, Conceptualization. **Taozhao Yu:** Methodology, Investigation. **Zhihua Yang:** Investigation. **Peter M. van der Kraan:** Writing – review & editing, Project administration, Funding acquisition. **Jeroen J.J.P. van den Beucken:** Writing – review & editing, Supervision, Resources, Methodology, Conceptualization. **Fons A.J. van de Loo:** Writing – review & editing, Supervision, Project administration, Methodology, Data curation, Conceptualization.

## Declaration of competing interest

The authors declare that they have no known competing financial interests or personal relationships that could have appeared to influence the work reported in this paper.

## Data Availability

Data will be made available on request.
