# Supplementary material for: Collagen binding properties separate two functionally distinct subpopulations of milk extracellular vesicles regarding bone regenerative capacity
Source: Mater Today Bio. 2025 Jul 18;33:102115. doi: 10.1016/j.mtbio.2025.102115 (PMC12302926; doi:10.1016/j.mtbio.2025.102115)
Supplement: Multimedia component 1 [file mmc1.docx]

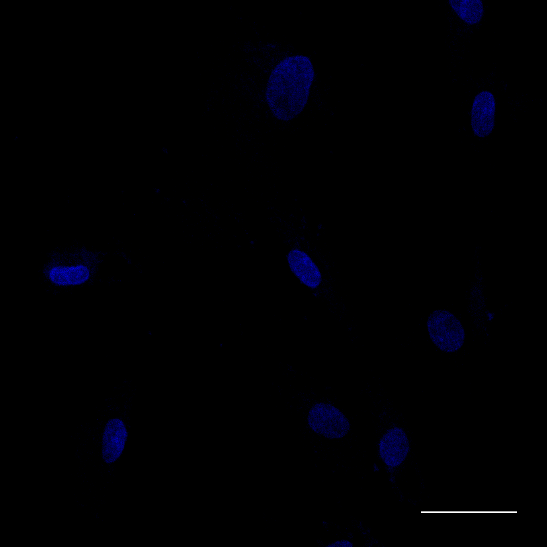


**Fig. S1.** Fluorescence image of dapi-stained hBMSCs cultured on collagen-coated tissue plastic without ^cb+^mEVs in 6h (Ctrl). Scale bars correspond to 100 μm.


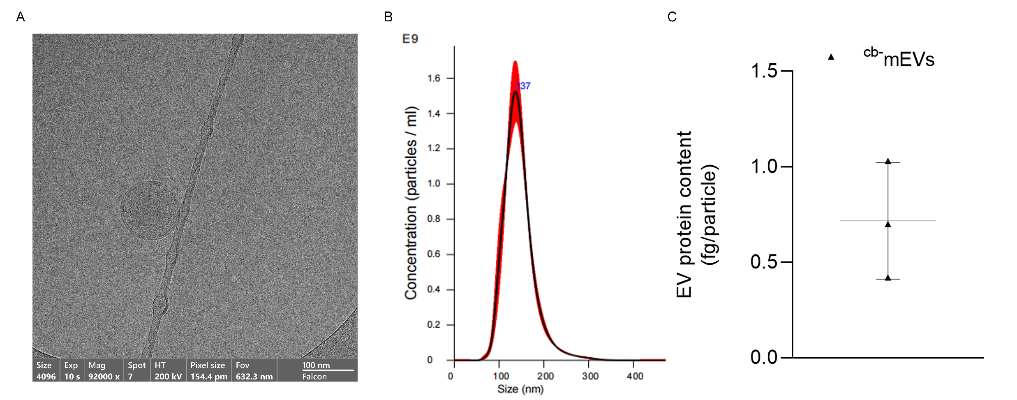


**Fig. S2.** ^cb-^mEVs characterization. A. Observation of the morphology of ^cb-^mEVs under Cryo-EM (92000x). B. NTA detection of the median particle size of ^cb-^mEVs (n=3). C. Determination of particle/protein ratio as ^cb-^mEVs protein content (n=3). Data were expressed as means ± standard deviation (SD).


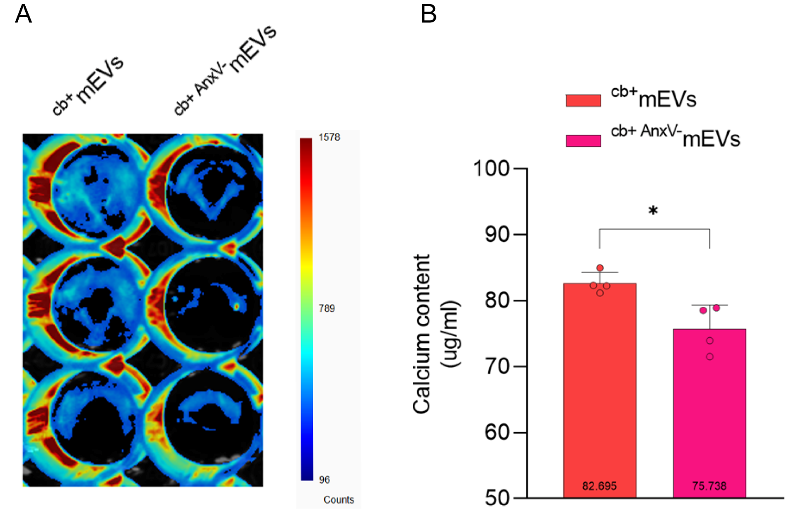


**Fig. S3**. Depleting AnxV diminished mEVs-collagen binding. A. IVIS image showing a representative image of the binding of fluorescently labeled ^cb+^mEVs and ^cb+ AnxV-^mEVs. B. Calcium content of hBMSCs cultured in OM in collagen-coated tissue culture plastic within ^cb+^mEVs or ^cb+ AnxV-^mEVs for 21 days (n=4). Data were expressed as means ± standard deviation (SD). *p < 0.05.
