# Supplementary material for: Collagen binding properties separate two functionally distinct subpopulations of milk extracellular vesicles regarding bone regenerative capacity
Source: Mater Today Bio. 2025 Jul 18;33:102115. doi: 10.1016/j.mtbio.2025.102115 (PMC12302926; doi:10.1016/j.mtbio.2025.102115)
Supplement: Multimedia component 2 [file mmc2.docx]

**Table S1.** List of proteins enriched in ^cb+^mEVs

| S.No | Protein accession | Gene symbol | Intensity mEVs-1 | Intensity mEVs-2 | Intensity ^cb-^mEVs-1 | Intensity ^cb-^mEVs -2 |
| --- | --- | --- | --- | --- | --- | --- |
| 1 | Q5E946 | PARK7 | 21.0 | 50.4 | 7.3 |  |
| 2 | P28783 | S100A9 | 36.0 | 24.7 | 7.6 |  |
| 3 | A0AAA9T9Y7 | CSN2 | 306.1 | 1479.5 | 167.4 | 283.6 |
| 4 | D3TJT8 | CSN1S1 | 233.9 | 17.8 | 34.4 |  |
| 5 | A5D7Q6 | PCDHGA2 | 98.3 | 166.5 | 47.4 | 46.3 |
| 6 | P02663 | CSN1S2 | 5850.5 | 441.7 | 2177.3 | 131.8 |
| 7 | Q28049 | LALBA | 91.4 | 91.7 | 28.2 | 43.8 |
| 8 | Q2UVX4 | C3 | 197.8 | 31.7 | 64.5 | 27.0 |
| 9 | F1N1Z8 | LOC104975830 | 255.1 | 105.2 | 118.9 | 37.3 |
| 10 | Q3SYR8 | JCHAIN | 2012.8 | 1329.6 | 1095.6 | 424.6 |
| 11 | Q8SPP7 | PGLYRP1 | 28.0 | 62.1 | 17.5 | 25.0 |
| 12 | F1N026 | ITM2B | 19.3 | 11.0 | 7.1 |  |
| 13 | P81265 | PIGR | 725.7 | 780.5 | 511.3 | 208.5 |
| 14 | P11151 | LPL | 728.7 | 624.3 | 361.2 | 288.3 |
| 15 | A4FV54 | RAB8A | 66.7 | 52.7 | 45.5 | 15.2 |
| 16 | Q862Q3 | B2M | 129.1 | 282.8 | 129.2 | 83.5 |
| 17 | Q0VBY4 | TPD52L1 | 8.0 | 17.0 | 6.7 |  |
| 18 | P30922 | CHI3L1 | 42.8 | 24.6 | 18.5 |  |
| 19 | D1Z306 | CRISPLD2 | 42.8 | 26.9 | 19.5 |  |
| 20 | E1BGX8 | HHIPL2 | 113.4 | 60.6 | 49.3 |  |
| 21 | Q95M40 | TXNDC17 | 29.6 | 18.9 | 20.8 | 6.9 |
| 22 | G3X6N3 | TF | 152.6 | 125.8 | 49.7 | 110.2 |
| 23 | Q0IIA4 | GP2 | 1092.1 | 244.4 | 726.2 | 80.7 |
| 24 | Q3ZCL0 | CRISP3 | 105.9 | 875.0 | 71.8 | 523.6 |
| 25 | F1MM32 | QSOX1 | 158.1 | 67.5 | 106.9 | 30.8 |
| 26 | F1N152 | HTRA1 | 15.7 | 11.3 | 8.4 |  |
| 27 | P05689 | CTSZ | 62.5 | 40.7 | 32.4 |  |
| 28 | P80195 | GLYCAM1 | 4661.2 | 1796.5 | 2441.8 | 1638.6 |
| 29 | P81287 | ANXA5 | 97.7 | 147.7 | 69.0 | 87.1 |
| 30 | Q2TBI0 | LBP | 51.7 | 15.4 | 21.6 |  |
| 31 | Q95JH2 | AGPAT1 | 65.5 | 50.5 | 37.4 |  |
| 32 | Q3ZCD0 | CD81 | 34.9 | 114.1 | 32.4 | 64.9 |
| 33 | Q148D9 | CREG1 | 37.8 | 15.5 | 17.5 |  |
| 34 | Q3T0D7 | SAR1A | 88.3 | 41.9 | 85.7 |  |
| 35 | P20072 | ANXA7 | 35.9 | 58.8 | 32.7 |  |
| 36 | Q32L41 | GCHFR | 69.3 | 10.0 | 41.2 | 14.2 |
| 37 | P80025 | LPO | 264.7 | 87.8 | 151.2 | 99.5 |
| 38 | A0A3Q1LGW7 | KRT76 | 5873.0 | 13577.5 | 4573.2 | 9889.2 |
| 39 | Q95114 | MFGE8 | 7021.0 | 1880.1 | 5397.8 | 1249.3 |
| 40 | F1N6Y1 | GANAB | 41.3 | 27.1 | 26.1 |  |
| 41 | G3N2D8 | GGT1 | 2843.6 | 3402.9 | 2601.1 | 2240.7 |
| 42 | P30932 | CD9 | 1446.3 | 198.6 | 1178.1 | 99.1 |
| 43 | Q5EA79 | GALM | 602.4 | 69.5 | 489.1 | 32.9 |
| 44 | Q3SZZ9 | FGG | 71.1 | 44.2 | 45.4 |  |
| 45 | Q5E994 | CHMP1B | 405.9 | 49.1 | 307.8 | 50.7 |
| 46 | Q8SPJ1 | JUP | 55.7 | 320.8 | 94.4 | 202.8 |
| 47 | Q0VCK0 | ATIC | 43.4 | 3.0 | 18.4 |  |
| 48 | A0AAA9T9N7 | PANK1 | 567.6 | 339.0 | 280.9 | 437.9 |
| 49 | Q2KJ22 | MINDY1 | 104.9 | 11.0 | 80.1 | 12.3 |
| 50 | F1MXH7 | CSF2RB | 46.7 | 23.0 | 28.1 |  |
| 51 | Q17QE5 | CIB1 | 325.3 | 144.2 | 275.0 | 104.2 |
| 52 | P00727 | LAP3 | 198.9 | 36.3 | 178.0 | 13.6 |
| 53 | E1BKT9 | DSP | 12.7 | 53.0 | 32.4 | 21.2 |
| 54 | Q32KV6 | SIL1 | 17.5 | 30.2 | 4.4 | 34.9 |
| 55 | Q8MJ50 | OSTF1 | 81.2 | 20.1 | 42.1 |  |
| 56 | Q3ZBD7 | GPI | 194.6 | 97.2 | 191.8 | 50.7 |
| 57 | A0AAA9TDJ4 | SDCBP | 101.0 | 19.5 | 57.8 | 43.2 |
| 58 | F1MNI4 | RAB5B | 72.0 | 29.6 | 78.7 | 6.7 |
| 59 | P50448 | SERPING1 | 134.8 | 82.0 | 81.1 | 101.8 |
| 60 | E1BP42 | CSAD | 36.1 | 17.3 | 22.6 |  |
| 61 | P50227 | SULT1A1 | 56.4 | 14.4 | 54.0 | 6.5 |
| 62 | Q4GZT4 | ABCG2 | 2891.3 | 1587.0 | 2175.1 | 1672.3 |
| 63 | Q3T000 | YKT6 | 202.1 | 55.9 | 192.3 | 32.0 |
| 64 | Q3T0U5 | CHMP2A | 435.1 | 51.4 | 352.1 | 77.4 |
| 65 | Q28161 | PKP1 | 31.3 | 47.0 | 34.6 |  |
| 66 | F1MYJ3 | GGT5 | 32.4 | 4.7 | 16.4 |  |
| 67 | A6QR35 | SCAMP2 | 74.5 | 20.7 | 80.9 | 3.7 |
| 68 | Q32KR9 | CHMP1A | 193.1 | 14.7 | 185.3 |  |
| 69 | Q27960 | SLC34A2 | 3688.2 | 758.6 | 3443.0 | 539.8 |
| 70 | Q3SZU5 | SPCS3 | 281.1 | 72.0 | 160.7 |  |
| 71 | A1L5C6 | HPN | 42.7 | 136.9 | 56.3 | 108.7 |
| 72 | P28782 | S100A8 | 224.0 | 9.0 |  |  |
| 73 | Q32L14 | RBP4 | 20.9 | 8.7 |  |  |

Intensity: Mass spectrometric signal intensity for a peptide is an indicator for its respective amount in the sample.
