# Supplementary material for: Collagen binding properties separate two functionally distinct subpopulations of milk extracellular vesicles regarding bone regenerative capacity
Source: Mater Today Bio. 2025 Jul 18;33:102115. doi: 10.1016/j.mtbio.2025.102115 (PMC12302926; doi:10.1016/j.mtbio.2025.102115)
Supplement: Multimedia component 3 [file mmc3.docx]

**Tables S2.** Defects for Histological Analysis in ROI

| Groups | Defects Created | Defects Included in Analysis |
| --- | --- | --- |
| Ctrl-col  mEVs-col  ^cb-^mEVs-col  ^AnxV-^mEVs-col | 18  6  6  6 | 17  6  6  5 |
